# Effects of Scheduled Employee Assistance Program Meetings on Resident Utilization of Counseling Services

**DOI:** 10.7759/cureus.108254

**Published:** 2026-05-04

**Authors:** Kyle Fletke, Leah S Millstein, Kristin Reavis, Christina Koch, Kathryn N Silva, Bernadette C Siaton, Erin Giudice, Katelyn E Donohue, Kimberly N Hollander, Stephanie Kahntroff, Stephanie Jones, Lawrence Reid, Danielle Baek

**Affiliations:** 1 Family Medicine, University of Maryland Medical Center, Baltimore, USA; 2 Internal Medicine, University of Maryland School of Medicine, Baltimore, USA; 3 Pediatrics, University of Maryland School of Medicine, Baltimore, USA; 4 Family Medicine, Kaiser Permanente, Washington DC, USA; 5 Anesthesiology, University of Maryland School of Medicine, Baltimore, USA; 6 Biostatistics, University of Maryland School of Medicine, Baltimore, USA

**Keywords:** employee assistance program, job satisfaction, provider burnout, resident resilience, wellness and resilience

## Abstract

Introduction: Employee assistance programs (EAPs) provide free and confidential counseling services to employees. This study will evaluate the impact of scheduled EAP sessions on the utilization of counseling services among residents across multiple subspecialties at an academic institution.

Methods: Prior to the study period, medicine, pediatrics, and medicine-pediatrics residency programs at a large academic institution were scheduling EAP appointments for the postgraduate year 1 (PGY-1) residents. During the 2023-2024 academic year, incoming PGY-1 residents from five residency programs (medicine, pediatrics, medicine-pediatrics, anesthesiology, and family medicine) were scheduled for an appointment with EAP. The PGY-1 residents completed a pre-EAP survey to evaluate EAP appointment perceived experiences. A post-EAP survey was distributed to all PGY-1 residents, upper-level residents who were not previously scheduled for EAP meetings (anesthesiology and family medicine), and to upper-level residents who had previously been scheduled for an EAP session as a PGY-1 (medicine, pediatrics, and medicine-pediatrics). Participants were also asked to complete the Physician Well-Being Index (PWBI).

Results: Incoming residents felt meetings with EAP would be beneficial if there was enough time to attend. Residents scheduled had a higher rate of attending at least a single EAP session (74%, N = 17) than those not scheduled (32%, N = 6) (p = 0.0119). The most common barriers to attending were relief from clinical duties and sufficient time off. There was no observed difference in the PWBI between scheduled and not-scheduled residents. This study was limited by multiple factors, primarily low statistical power and a low response rate to both the pre- and post-EAP surveys.

Conclusions: Residency programs across different specialties may increase attendance to at least a single EAP session by scheduling residents and providing relief from clinic duties with sufficient time off. Further studies are required to determine the impact of scheduled EAP sessions on resident well-being.

## Introduction

Residency training can be physically and emotionally demanding and is associated with resident well-being that is often below population norms [1-3]. Interventions that have been observed to potentially enhance well-being and reduce burnout include sleep, time away from work, individual coaching, meditation, and strong social relatedness [2,4]. Current literature addresses various methods to facilitate resident awareness and access to these supportive interventions, including well-being assessments and automatic enrollment in wellness assessments or counseling sessions [5-8]. A universal, systems-based approach to incorporating wellness into residency program systems, rather than relying on the identification of individuals perceived to be in need, has been supported in the literature [1,9]. Many residency programs have access to employee assistance programs (EAPs), which provide free counseling and other professional services to employees. Residents who are scheduled for EAP sessions have more positive perceptions of EAP and are more willing to engage in future mental health services [7-8]. However, limited time to attend appointments and concerns regarding confidentiality are known barriers to resident use of EAP services [2]. Residency programs can increase utilization of EAP services by scheduling dedicated sessions [7,8,10]. 

The University of Maryland Medical Center (UMMC) has an established EAP department available to residents. Multiple residency programs (internal medicine, pediatrics, and medicine-pediatrics) have previously scheduled all postgraduate year 1 (PGY-1) residents for introductory EAP sessions. These sessions followed an opt-out model, whereby appointments were automatically scheduled, and residents could elect to cancel. During the 2023-2024 academic year, family medicine and anesthesiology scheduled similar opt-out sessions. This study will compare the use of EAP services among residency programs that have utilized opt-out scheduling with those that previously used a resident-driven self-scheduled approach. A secondary outcome that will be evaluated is the impact of scheduled EAP sessions on resident well-being. 

## Materials and methods

Prior to the study period, three residency programs at the UMMC (internal medicine, pediatrics, and medicine-pediatrics) were offering opt-out EAP appointments to residents within the first six months of residency training. The anesthesiology and family medicine residency programs did not have a prior existing program in place for EAP appointments. While family medicine and anesthesiology residents had the opportunity to attend the institution's EAP, they were not scheduled by the program, and protected time was not made available to attend. 

During the 2023-2024 academic year, PGY-1 residents (n = 75) from all five residency programs (medicine, family medicine, anesthesiology, medicine-pediatrics, and pediatrics) were scheduled for an opt-out appointment with EAP during the first four months of their residency training. Appointment times were coordinated between residency program leadership and the EAP administrative staff. Residents were informed of their scheduled time and advised that the time was protected from work responsibilities. Additional appointments were scheduled by the residents as requested. Residency programs were not informed of resident attendance at the EAP sessions. Confidential information obtained during these sessions was not shared with residency program leadership. EAP appointments are not recorded in the institution's electronic medical record. 

All PGY-1 residents were invited to complete a pre-EAP survey in June 2023 prior to their first scheduled session. No residents were excluded. The pre-EAP survey was created by the study team utilizing existing literature about EAP utilization among resident physicians [7,8]. The survey was not assessed for validity. This survey collected demographic information, data on prior use of counseling or professional services through an employer or independent provider, interest in attending EAP sessions, and perceived barriers to attending such sessions. 

Post-EAP survey

Starting in September 2023, residents from all five programs in all PGY years (inclusive of PGY-1 residents who completed the pre-EAP survey) (n = 249) were invited to participate in an anonymous post-EAP survey. The post-EAP survey was created by the study team utilizing existing literature about EAP utilization among resident physicians [7,8]. The survey was not assessed for validity. No residents were excluded. Two separate versions of the post-EAP survey were distributed to account for the different program histories of scheduling EAP appointments. 

Not Scheduled

This is offered to upper-level residents not scheduled for EAP meetings in their PGY-1 year (family medicine and anesthesiology, n = 50). 

Scheduled

This is offered to any resident who had been scheduled to attend an EAP session in their first year (all PGY 1-4 medicine, pediatrics, medicine-pediatrics, and PGY-1 family medicine and anesthesiology, n = 199). 

In addition, all respondents were asked to complete the Physician Well-Being Index (PWBI), a validated tool to evaluate healthcare professionals' well-being [11-14]. Permission to utilize the PWBI for research purposes can be obtained through Corporate Web Services, Inc. [15]. 

Informed consent to participate in the study was obtained for each participant prior to each of the surveys. This study met the definition of Not Human Subjects Research and did not require IRB oversight as determined by the IRB of the University of Maryland, Baltimore. 

## Results

Pre-EAP survey for PGY-1 residents 

A total of 33 residents (44% response rate) completed the survey. Characteristics of the participants are outlined in Table 1. Nineteen participants had previously utilized counseling services through an employer (n = 5) or an independent provider (n = 14). There was no difference in prior counseling rates between the residency programs (p = 0.18). Most residents (n = 23, 70%) somewhat agreed or strongly agreed that meetings with EAP would have a beneficial role in their development. The most common anticipated barrier for attending an EAP meeting was time to attend meetings/relief of clinical duties (n = 30, 90%), followed by concern for confidentiality (n = 13, 39%), and potential to be identified as less competent (n = 11, 33%), with most respondents citing multiple potential barriers. 

**Table 1:** Characteristics of study participants *Other race includes Native Hawaiian, Hispanic, and other race/ethnicity not listed

		Pre-survey	Post-scheduled	Post-not scheduled
Total N (%)		33 (100)	23 (100)	19 (100)
Age (years)	25-29	24 (72.7)	14 (60.9)	10 (52.6)
	30-34	6 (18.2)	8 (34.8)	4 (21.1)
	35-39	2 (6.1)	1 (4.4)	4 (21.1)
	Prefer not to answer	1 (3.0)	0 (0.0)	1 (5.3)
Gender	Female (cisgender)	24 (72.7)	21 (91.3)	9 (47.4)
	Male (cisgender)	7 (21.2)	1 (4.4)	7 (36.8)
	Nonbinary/3rd gender	1 (3.0)	1 (4.4)	1 (5.3)
	Transgender male	0 (0.0)	0 (0.0)	1 (5.3)
	Prefer not to answer	1 (3.0)	0 (0.0)	1 (5.3)
Race	White/Caucasian	16 (48.5)	14 (60.9)	7 (36.8)
	Black/African American	3 (9.1)	3 (13.0)	4 (21.1)
	Asian/Asian American	6 (18.2)	5 (21.7)	2 (10.5)
	Other*	5 (15.2)	1 (4.4)	3 (15.8)
	Prefer not to answer	3 (9.1)	0 (0.0)	3 (15.8)
Residency Program	Pediatrics	14 (42.2)	17 (73.9)	2 (10.5)
	Family medicine	7 (21.2)	5 (21.7)	10 (52.6)
	Internal medicine	9 (27.3)	1 (4.4)	0 (0.0)
	Anesthesiology	2 (6.1)	0 (0.0)	6 (31.6)
	Prefer not to answer	1 (3.0)	0 (0.0)	1 (5.3)

Post-EAP survey results (scheduled and not scheduled) 

A total of 42 residents (23 scheduled during PGY-1 year, 19 not scheduled during PGY-1 year; 17% response rate) completed the post-EAP surveys. Characteristics of the participants are outlined in Table 1. Residents scheduled for an EAP visit had a higher rate of attending at least a single EAP session (74%, n = 17) than those not scheduled (32%, n = 6) (p = 0.01, Fisher's exact test). The proportion of residents who had no, one, or multiple EAP session meetings significantly differed for scheduled (26%, 61%, 13%, respectively) and not-scheduled (68%, 21%, 11%, respectively) residents (Fisher's exact p = 0.015). No difference was identified between the groups for multiple sessions attended. 

The most common service sought during EAP visits was self-resiliency/self-care. Anxiety/depression/other mood disorder, compassion fatigue, and grief support were the next most common services obtained. The most common barriers to attending meetings were relief from clinical duties and sufficient time off/PTO (n = 24 and n = 23, respectively). The perceived social stigma of attending mental health counseling (n = 6) was another common barrier. 

Half of the residents who had never been scheduled for an EAP visit did not know how to schedule an EAP meeting, compared to only 21% of previously scheduled residents. Most residents (91% scheduled and 75% not-scheduled, respectively) recommended that future residents have EAP meetings scheduled by the residency program. 

There was no difference in PWBI scores between scheduled and not-scheduled respondents (p = 0.55; Table 2). Among scheduled residents, no difference in mean PWI scores was observed between those who attended versus did not attend their first scheduled EAP meeting (p = 0.17). Job satisfaction was not different among residents who were scheduled for an EAP appointment compared to those who were not scheduled (p = 0.78). 

**Table 2:** Mean PWBI scores by resident EAP scheduling status PWBI: Physician Well-Being Index; EAP: employee assistance program; SD: standard deviation Interpretation of PWBI scores for resident physicians can be found in the article by Drybye et al. [11]. SD (95% CI) refers to the 95% CI for the standard deviation of the PWBI scores

	N	Mean (SD)	SD (95% CI)
Overall	42	3.69 (1.96)	(3.08, 4.30)
Not scheduled	19	3.52 (1.75)	(2.76, 4.28)
Scheduled	23	3.89 (2.21)	(2.83, 4.96)
Attended 1st EAP mtg	17	3.82 (1.85)	(2.87, 4.77)
Did not attend 1st EAP mtg	6	2.67 (1.21)	(1.40, 3.94)

## Discussion

This study demonstrated that most incoming residents representing five residency programs at a large academic medical institution felt that meetings with EAP would be beneficial to their professional development and well-being. Multiple perceived barriers to attending these sessions were identified, including potential lack of protected time to attend these meetings, concern for lack of confidentiality, or perception of being less competent than their peers. These findings are consistent with prior studies [2]. 

Residents with program-scheduled appointments were more likely to attend an initial EAP session. However, many potential barriers exist to scheduling an appointment with EAP, with a perceived lack of protected time to attend appointments as the greatest barrier. Therefore, residency programs can look to increase attendance to at least a single EAP session by scheduling a meeting time for the residents and providing relief from clinical duties. Each program in this study utilized various strategies to provide protected time. For example, family medicine scheduled the EAP meetings on dedicated days during orientation, in which no other activities or clinical duties were planned. Pediatrics scheduled the sessions during half-day periods when other clinical responsibilities were not expected. 

There was no difference in the percentage of residents who attended multiple sessions between the scheduled and not-scheduled groups. The reason for this finding was not identified; however, it can be speculated that this is related to the multiple identified barriers to scheduling an appointment. One barrier is a lack of understanding of how to schedule an EAP visit. Residents who were scheduled for an initial EAP visit became more familiar with the process for arranging appointments; however, there remained a large percentage of residents who were still unfamiliar with the process. Another commonly cited barrier to EAP utilization among residents is concern regarding confidentiality. During the initial EAP session, residents are informed about their EAP benefits, including access to multiple, free, trauma-informed counseling sessions. Confidentiality policies are reviewed, and residents complete a consent form prior to initiating services. This discussion may help alleviate concerns that information disclosed during EAP sessions could be shared with residency leadership. 

During the initial EAP session, residents also receive brief education on the importance of self-care and burnout prevention during residency. In addition, the EAP team assesses existing mental health concerns, explores prior coping strategies, and facilitates referral to additional mental health services when indicated. Although no difference in physician wellness, as measured by the PWBI, was detected between residents who were scheduled for an EAP session and those who were not, residents who attended an initial session were nonetheless introduced to the concept of wellness. The absence of a difference in PWBI scores is likely influenced by multiple factors. Although not directly measured in this study, engagement in independent mental health services, personal life stressors, and variations in the clinical work environment may have contributed to this finding. 

This study was limited by multiple factors, primarily low statistical power and a low response rate to both the pre- and post-EAP surveys. A comparison between participants in different residency programs in PWBI and overall job satisfaction could not be determined due to the low response rate. Additionally, the resident respondents in the "not scheduled" and "scheduled" groups differed in training year and specialty, limiting the comparisons that can be made between these groups strictly on scheduled status. Future studies should enroll a larger sample population to improve the study's overall power. Residency programs could also provide dedicated time to answer survey questions to remove the burden of having to complete the surveys and the PWBI. Stigma associated with attending mental health sessions was identified as a potential barrier. Future studies may look to utilize a validated tool, such as the Mental Health Service Stigma Assessment Questionnaire (MHS-SAQ), to evaluate whether scheduled EAP appointments help to lower the stigma related to attending mental health sessions. 

## Conclusions

Residency programs can utilize already established EAPs within their institutions as part of a broader effort to support resident well-being. Although a difference in well-being as identified by the PWBI was not identified in this study, residency programs across different specialties can increase initial engagement with EAP by scheduling residents for an initial appointment and providing relief from clinic duties. Structured use of existing EAP resources may help normalize engagement with mental health services during residency training while offering a feasible approach to supporting resident wellness. Further studies are required to determine the impact of scheduled EAP sessions on resident well-being. Such studies could look to enroll a larger sample size across multiple institutions with established EAP resources and follow residents throughout the course of their training. 
